# Eggshell Types and Their Evolutionary Correlation with Life-History Strategies in Squamates

**DOI:** 10.1371/journal.pone.0138785

**Published:** 2015-09-22

**Authors:** Konstantin Hallmann, Eva Maria Griebeler

**Affiliations:** Department of Evolutionary Ecology, Institute of Zoology, Johannes Gutenberg-University Mainz, Rhineland-Palatinate, Germany; Universidad de Granada, SPAIN

## Abstract

The eggshell is an important physiological structure for the embryo. It enables gas exchange, physical protection and is a calcium reserve. Most squamates (lizards, snakes, worm lizards) lay parchment-shelled eggs, whereas only some gekkotan species, a subgroup of lizards, have strongly calcified eggshells. In viviparous (live-bearing) squamates the eggshell is reduced or completely missing (hereafter “shell-less”). Recent studies showed that life-history strategies of gekkotan species differ between species with parchment- and rigid-shelled eggshells. Here we test if the three different eggshell types found in the squamates are also associated with different life-history strategies. We first investigated the influence of the phylogeny on the trait “eggshell type” and on six life-history traits of 32 squamate species. Phylogenetic principal component analysis (pPCA) was then conducted to identify an association between life-history strategies and eggshell types. Finally, we also considered adult weight in the pPCA to examine its potential effect on this association. Eggshell types in squamates show a strong phylogenetic signal at a low taxonomical level. Four out of the six life-history traits showed also a phylogenetic signal (birth size, clutch size, clutches per year and age at female maturity), while two had none (incubation time, maximum longevity). The pPCA suggested an association of life-history strategies and eggshell types, which disappeared when adult weight was included in the analysis. We conclude that the variability seen in eggshell types of squamates is weakly influenced by phylogeny. Eggshell types correlate with different life-history strategies, and mainly reflect differences in adult weights of species.

## Introduction

The advent of the first amniotes about 315 to 355 million years ago marked the transition from an exclusively water-dependent reproduction to the capability of a water-independent reproduction in drier environments, representing one of the most important events of evolution [1]. A precondition for this was the development of the amniotic egg characterized by additional extra-embryonic membranes, which made embryogenesis independent from water. The amniotic egg shows characteristic taxonomic features such as an at least partially mineralized eggshell, which is an autapomorphy of *Sauropsida* (the extant members of which are the turtles, lepidosaurs, crocodiles and birds; [2]).

The eggshell is an important structure, linking internal physiological processes to the ambient environment. As an adaption to exterior conditions, the eggshell exhibits specific characteristics such as permeability, which allows gas exchange via diffusion and the uptake of certain amounts of water [3]. Additionally, the eggshell physically protects the developing embryo, reducing the risk of egg damage during oviposition and defending it against attacking arthropods or other predators after oviposition [3]. A calcareous shell matrix is also a calcium reserve for the development of the embryo besides its physical protection [4,5].

The eggshell of most squamates (lizards, snakes, amphisbaenians = “worm lizards”) has a simple and uniform structure, consisting of a thick shell membrane of protein fibrils, which adds to the flexibility of the egg [3]. On top, the shells can be superficially calcified [3]. Apart from this, the eggs of a subgroup of lizards, the *Gekkota*, have a very specialized and divergent shell structure. Their strongly calcified shell consists of three layers: a reticular boundary layer, a thin but dense shell membrane and a thick calcareous layer [3]. In the latter the calcite crystallites are irregularly arranged.

Besides oviparous (egg-laying) species, there are more than 100 independent origins of viviparity (live-bearing) within the squamates [6–8], which makes this group a perfect choice for studying the evolution of viviparity in general. Viviparity involves egg retention and a reduced eggshell. Most squamates show lecithotrophic viviparity [9–11], where nutrients are supplied mostly by the yolk sac. Only few squamates show matrotrophic viviparity [10,12], where nutrients are supplied via maternal placenta-like structures. Irrespective of these differences in the nutrition of the embryo, in squamates viviparity is associated with only a vestigial remnant of the eggshell, thus the species have “shell-less eggs” [13].

The interest in the evolution of life-history and reproductive strategies of lizards and snakes, and in general of squamates, started with some comparative studies in lizards [14,15]. These studies provided first references for different reproductive strategies among lizards: early maturing and multiple-brooded vs. late-maturing and single-brooded. In the early-maturing group oviparity is almost universal, while viviparity is one form of the late-maturing strategy [15]. Dunham et al. [16] confirmed these two strategies for lizards, and they also discovered different life-history strategies within snakes for the reproduction modes.

Recently a study examined the relevance of eggshell types for the evolution of life-history strategies in the lizard group *Gekkota* [17]. Pike et al. [17] considered body length as a covariable in the analysis and were able to show, that life-history strategies (egg volume, hatchling size, developmental stage at oviposition, incubation duration) in *Gekkota* are actually related to the structure of their eggshells. For example, gekkotan species laying rigid-shelled eggs lay smaller eggs relative to adult body length and exhibit a smaller adult body length than species laying parchment-shelled eggs [17]. However, that study only aimed at a small taxonomic group, and information on life-history traits besides body length was limited. For that reason, we considerably broadened the taxonomic scope in our analyses and used information on more life-history traits to test whether life-history strategies are linked to eggshell types in squamates as hypothesised by Pike et al. [17] only for the lizard subgroup *Gekkota*. Squamates as a whole represent a highly heterogeneous group in terms of life-style (nocturnal/diurnal, aquatic/arboreal/terrestrial), body size (e.g., 16 mm for the dwarf gecko *Sphaerodactylus ariasae* to 10 m as seen in the python *Malayopython reticulatus*) and geographic distribution (worldwide; except for Antarctica). The broader taxonomic approach required to take into account the shell-less type as a third eggshell type (beside the rigid-shelled and parchment-shelled types studied by Pike et al. [17]). Life-history traits included in our analysis for life-history strategies covered different phases during the life of an animal, spanning from the incubation of the egg to the adult individual. Thus, our approach is based on Pike et al. [17], but it goes considerably beyond that study.

First, we examined in our study whether eggshell types and studied life-history traits of species are actually influenced by their shared evolutionary history. In particular, we aimed at the following questions: Is the potential phylogenetic signal on eggshell types limited to the species level, or is it even present at higher taxonomic levels? What are the evolutionary transition rates between the different eggshell types?

Second, to explore whether eggshell types and life-history strategies (values of life-history traits and their combination) of species are actually associated, we used different statistical methods for data classification and ordination, which trace back to some classical comparative studies [15,16]. We expected, under the hypothesis of eggshell types being important in the evolution of squamate life-history strategies, that species clustering together because of similar life-history traits would share the identical eggshell type. We conducted further analyses to clarify whether in squamates the shared evolutionary history, or adult body mass are main drivers of the clustering seen in life-history strategies and their association to egg shell types.

## Materials and Methods

### Life-history data

To test whether eggshell types are linked to different life-history strategies in squamates we mainly focused on age-related, reproductive and size-related life-history traits: age at maturity (days), adult weight (g), birth size (total length, cm), clutch size, number of clutches per year, incubation time (days) and maximum longevity (years). We selected adult weight instead of body length or snout-vent-length as a measure of animal size because of the great diversity of body shapes seen in squamates. To account for differences in the geographic distribution of species and their potential influence on the life-history traits of species, we also collected information on the maximum altitude (m) of areas inhabited by species [18–21]. We chose only altitude and did not consider latitude because different studies on the squamate genera *Phrynosoma* and *Sceloporus* demonstrated that altitudinal variation is much more important for the evolution of viviparity (shell-less eggs) and the reproductive mode in general than latitudinal variation [21–23].

Life-history data of species and maximum altitude were mainly taken from the multivolume encyclopedia on European reptiles, the “Handbuch der Reptilien und Amphibien Europas” [24–30]. We also used the internet database AnAge for worldwide distributed squamate species [31] to fill in the geographic and phylogenetic gaps of the encyclopedia. Unfortunately, this database primarily focuses on age-related life-history traits, such as maximum longevity and age at first maturity. We therefore further conducted an intensive literature search comprising primary literature [32–35], field guides, textbooks and, only for the geckos (*Gekkota*), specific literature [e.g. 35–39]. To ensure a sufficient standard of data quality, we did not consider anecdotal remarks given in these sources. In the cases we found ranges or multiple values for species traits we always averaged these for statistical analyses. This procedure in particular prevented a gender bias in incubation time values, because sex-determination in squamate reptiles is based on temperature [40].

Information on maximum altitude of species was completed by estimates found in different papers [41–46].

In total, we collected data on the aforementioned seven life-history traits of 574 squamate species. The information on life-history traits and adult weight was not evenly distributed on the 574 squamate species. For multivariate analyses, this resulted in a substantial reduction of sample size to 32 squamate species. To address the issue that sample size is low, we re-examined our results by repeating analyses for a dataset with an order of magnitude more squamate species (n = 300) but comprising fewer traits (n = 5) from a recent publication ([47]; see discussion for further details). With this larger dataset we were also able to address a potential geographic bias in the distribution of eggshell types within our dataset on 32 squamates. The dataset of Scharf et al. [47] comprises worldwide distributed squamate species.

### Classification of eggshell types

Although there is high structural variability in eggshells of squamates, we assigned species to only three types in order to study the association between eggshell types and life-history strategies (but see Pike et al. [17]). However, Schleich and Kästle [3] pointed out: “While it is relatively easy to characterize shells of different orders, it is very difficult to do so at the family or species level, as for instance in squamate shell surfaces.” The reasons for this are the similarity of eggshells of phylogenetically very distant forms and the extreme variability of organic and inorganic shell elements [3]. Nevertheless, we think, it is possible to classify eggshell types within the *Squamata*, if we relax the definition of the term “eggshell”. Packard et al. [48] used the term eggshell as “a generic term referring to all layers of a freshly laid egg external to the albumen”. For the squamates this implies two eggshell types: (1) a 2-layered parchment shell, consisting of a boundary layer and a fibrous shell membrane, which is the most common shell type of the oviparous squamate species; (2) a strongly calcified 3-layered shell with a reticular boundary layer, a dense fibrous shell membrane and a calcareous shell layer, which can only be found in a subclade of *Gekkota* (*Gekkonidae*, *Phyllodactylidae* and *Sphaerodactylidae*) [2,3,17,49,50]. However, Pyron and Burbrink [51] recently characterised the parity mode of 8006 of the ~ 9400 known extant species, and assigned 1336 species to the parity mode “viviparity”, which comprised real viviparity and ovo-viviparity (sometimes called vivi-oviparity, see Lodé [52] for details; nutrients mostly provided by yolk). Due to the large number of viviparous squamates, we think that there is no compelling reason to ignore all those species, which share the feature of having “no” eggshell [13]. We therefore considered in our analysis a further eggshell type, and thus, a putatively third life-history strategy, which comprises all the live-bearing species, regardless of the way oxygen and nutrition is provided to the embryo. Characteristic for this “shell-less” eggshell type is the absence of a real eggshell defined by the existence of a boundary layer, a fibrous shell membrane and, in some rare cases, an additional calciferous shell layer. All shell-less species of our dataset were ovoviviparous. Thus, there was no systematic bias because of the broad concept of the term “shell-less”.

We gathered information on eggshell types for all 574 squamate species through an extensive literature search and from previous synopses [3,17,51,53,54]. We assigned each squamate species to a particular eggshell type: parchment-shelled, rigid-shelled or shell-less. For the common lizard *Zootoca vivipara*, for which both oviparous and viviparous forms exist, we assumed the shell-less type in our analyses, because of the considerably higher frequency of viviparous than oviparous animals found in the field [55,56]. Our classification revealed that most of the finally studied 32 squamate species belonged to the parchment-shelled type (18 species), and in equal proportions (7 species) to the rigid-shelled and shell-less type.

### Data analyses

All statistical analyses were performed with the statistical software R v3.02 [57] and additional packages (see below) available for this software. Initially, we tested for correlations between maximum altitude and life-history traits of species, by taking into consideration the effects of body mass. We therefore established phylogenetic generalized least square fit models (PGLS) for each of the life-history traits with maximum altitude and adult weight as independent variables using the function *gls* of the R-package *nlme* [58]. As the phylogenetic correlation matrix, we used the large-scale squamate phylogeny of Pyron and Burbrink [51].

#### Phylogenetic signal

A phylogenetic signal is present in species traits, because traits characterize species and species are related by their phylogeny [59]. To test for phylogenetic signals in egg shell types and studied life-history traits (age at maturity, birth size, clutch size, number of clutches per year, incubation time and maximum longevity) we always generated the respective phylogenetic tree for the squamate dataset studied by pruning an already published phylogeny, which is based on an ultrametric maximum likelihood tree of 4161 squamate species [51,60].

#### Eggshell type

In all analyses the discrete trait eggshell type was coded as follows: 1 = shell-less, 2 = parchment-shelled, and 3 = rigid-shelled. To estimate the strength of the phylogenetic signal in the discrete trait “eggshell type” for our dataset consisting of 32 species, we used comparative statistics for this trait that are based on the maximum likelihood approach. We initially applied the function *transform*.*phylo* from the R-package *geiger* [61], which effectively transforms the tree so that the scaling parameter λ fits the extent to which the phylogeny predicts the covariance among trait values for species. The resulting transformed tree indicates the strength of the phylogenetic structure: *λ* = 0 leads to a star-like tree with long terminal branches, indicating no phylogenetic structure of the observed trait values; *λ* = 1 corresponds to the original tree with untransformed branch lengths, thus recovering the Brownian motion (BM) model of evolution. From this approach we obtained the three trees assuming three λ values 0, 0.5 and 1 (original tree).

We then used the function *fitDiscrete* to establish continuous-time Markov models of discrete trait evolution based on these three trees considering different models of character states (all-rates-different, equal rates, meristic) in order to estimate transition rates between eggshell types. The all-rates-different (ARD) model allows different rates for each transition between two eggshell types (e.g. from eggshell type 1 to 2, but also from 2 to 1 etc.). The equal rates (ER) model assumes a single value for all transition rates between eggshell types. The meristic model allows only stepwise transitions between eggshell types. A direct transition from shell-less to rigid-shelled and vice versa was forbidden [61]. To identify the best of the nine models in terms of trait evolution (ARD, ER, meristic) and phylogeny (λ = 0, 0.5, 1) we compared their goodness of fit by using the Akaike information criterion (AIC) and the small-sample-size corrected AIC (AICc) [62,63].

We additionally used the function *fitDiscrete* with a lambda-transformation to estimate and optimize the λ values by maximum likelihood optimization given different character state models (ARD, ER, meristic) and an untransformed tree (equal to lambda λ = 1, Table 1). With this approach the evolutionary model of eggshell types is testable.

**Table 1:** Results of continuous-time Markov models of discrete trait evolution for the trait eggshell type for 32 squamate species.

Character states models	Lambda	AIC	AICc	q_12_	q_13_	q_21_	q_23_	q_31_	q_32_
ER	0	65.27	65.41	-	-	-	-	-	-
ARD	0	75.27	78.77	-	-	-	-	-	-
meristic	0	67.27	67.70	-	-	-	-	-	-
ER	0.5	47.36	47.49	-	-	-	-	-	-
ARD	0.5	53.01	56.51	-	-	-	-	-	-
meristic	0.5	45.40	45.83	-	-	-	-	-	-
ER	1.0*	42.51	42.93	0.0017	0.0017	0.0017	0.0017	0.0017	0.0017
ARD	1.0*	47.94	52.81	0.0119	0.0023	0.0043	<0.0001	<0.0001	0.0017
meristic	1.0*	40.76	41.65	0.0036	Fixed(0)	0.0036	0.0010	Fixed(0)	0.0010

Shown are models of discrete trait evolution that assume different character states models (ER = equal rates, ARD = all-rates-different, meristic = stepwise transition between countable traits) and different lambda (λ) values (star-like tree with *λ* = 0; original tree with *λ* = 1). Additionally presented are the model likelihoods based on the Akaike Information Criterion (AIC) (AICc = sample-size-corrected AIC). Models with an untransformed phylogenetic tree (original tree) provided the best AIC values for the different models of character states (ER, ARD, meristric). Only for these models the transition rates (q_nm_ with subscripts for eggshell types; 1 = shell-less, 2 = parchment-shelled, 3 = rigid-shelled) from one eggshell type to another are shown. *The λ value was additionally estimated and optimized by the *fitDiscrete* function (see main text). In the case of the meristic trait model, a direct transition from shell-less to rigid-shelled and vice versa is impossible (q_13_ or q_31_ = 0).

To determine and visualize the phylogenetic autocorrelation at different taxonomic levels (genus, family, infraorder/superfamily) for the trait eggshell type, we calculated phylogenetic correlograms [64] using our dataset with the 32 squamate species but also the complete dataset with 574 species. Gittleman and Kot [64] suggested the use of those correlograms to visualize the results of phylogenetic autocorrelative analyses, but also to compare the correlation at different distance categories [59]. Following these authors, we computed the correlation coefficient (Moran's I) at the genus level among pairs of species belonging to the same genus and for two higher taxonomic levels (family, infraorder/superfamily). We therefore used the function *correlogram*.*formula* from the R-package *ape* [65]. The allocation of species to the higher taxonomic groups followed the taxonomy of the Integrated Taxonomic Information System (ITIS), which is also used in the database AnAge [31]. For the highest taxonomic level studied, we merged the taxonomic levels infraorder and superfamily. We derived information on species membership to these two levels from "The Reptile Database" [66].

#### Life-history traits

To identify the phylogenetic autocorrelation of quantitative life-history traits among species, we used the function abouheif.moran as implemented in the R-package *adephylo* [67]. It is a multivariate, nonparametric procedure based on Moran’s I for detecting phylogenetic signals in traits by performing independent Monte Carlo tests for each trait analysed. As a measure of phylogenetic similarity we used the "oriAbouheif" proximity, which is based on Abouheif's test for serial independence (TFSI) and provides a mean C-statistic [68]. With this approach we were able to compare phylogenetic patterns seen in some life-history traits to those seen in the trait “eggshell type” for the species from our squamate dataset.

#### Methods for classification and ordination: cluster analysis and phylogenetic principal component analysis (pPCA)

To explore if species having a particular eggshell type share the same life-history strategy (in terms of the other traits) we first carried out a classical statistical approach [15,16]. We standardized the distribution of trait values for each life-history trait to a mean of zero and unit variance to ensure that each trait contributed equally to the dissimilarity matrix. We conducted a cluster analysis based on a dissimilarity matrix established from life-history traits of species to create a phenogram. Its coefficients of dissimilarity were calculated as *d*_*ij*_ = ∑_*i*,*k*_(*X*_*ik*_ − *X*_*jk*_) where *X*_*ik*_ is the state of the *k*th trait of species *i* and *X*_*jk*_ is the state of the *k*th trait of species *j* [15]. For the clustering process, we used the function *hclust* as implemented in the package *vegan* [69] that applies the nearest neighbour algorithm as clustering algorithm, which is closely related to the algorithm used to construct a minimal spanning tree [70]. We transformed the resulting dendrogram into a phylogram with the function *as*.*phylo* from the package *ape* [65]. To compare it to the respective pruned phylogenetic tree from a recently published squamate phylogeny [51], we used the function *compare*.*phylo* from the R-package *phyloch* [71]. The function *compare*.*phylo* compares the topological arrangements of two ultrametric trees based on branching times [59]. Thus, we could explore if the resulting clusters derived for life-history traits of species (or life-history traits and adult weight of species) coincide with the phylogeny or if species sharing the same eggshell type cluster together based on similar life-history trait values (or similar life-history trait and adult weight values). A comparison of a cluster analysis and a principal component analysis (PCA) can explain or confirm differences between groups of objects [72]. We therefore next conducted phylogenetic principal component analyses (pPCA) by using the function *ppca* from the R-package *adephylo* [67]. The phylogenetic proximity matrix between trait vectors of species was computed as implemented in the method "oriAbouheif" of the function *ppca* [73]. We again first standardized the dataset to a distribution with a mean of zero and unit variance to ensure equal contribution of the traits. For evaluation of the pPCA we selected the principal component with the largest (i.e., most positive) eigenvalue, indicating global structures (close-to-root variation in trait states) and the principal component with the lowest (i.e., most negative) eigenvalue, indicating local structures (close-to-tips variation in trait states) [74]. To correct for the effect of body mass on life-history traits in the pPCA, we additionally established univariate linear regression models for each life-history trait with adult weight as the predictor variable [74]. We repeated the pPCA using only the residuals from these linear models for species traits, to compare this weight-adjusted pPCA with a non-weight-adjusted pPCA. To test for significant differences between the clustering of the eggshell types, we conducted MANOVAs using the scores of PC1 and PC2 obtained from the pPCAs as dependent variables and the eggshell types as grouping factor. This was done with the function *manova* from the R-package *stats* [57].

## Results

The difference in the geographic distribution of the species as assessed by maximum altitude had no significant effect on any of the life-history traits of the squamates in our dataset (see S3 Table). Multiple regression analyses using maximum altitude and adult weight as predictors of life-history traits indicated only a significant influence of adult weight on some of the life-history traits. Thus, maximum altitude was excluded from our further analyses.

### Phylogenetic signal

#### Eggshell type

There was a clear phylogenetic signal for the trait “eggshell type” (Table 1).

**Fig 1:**
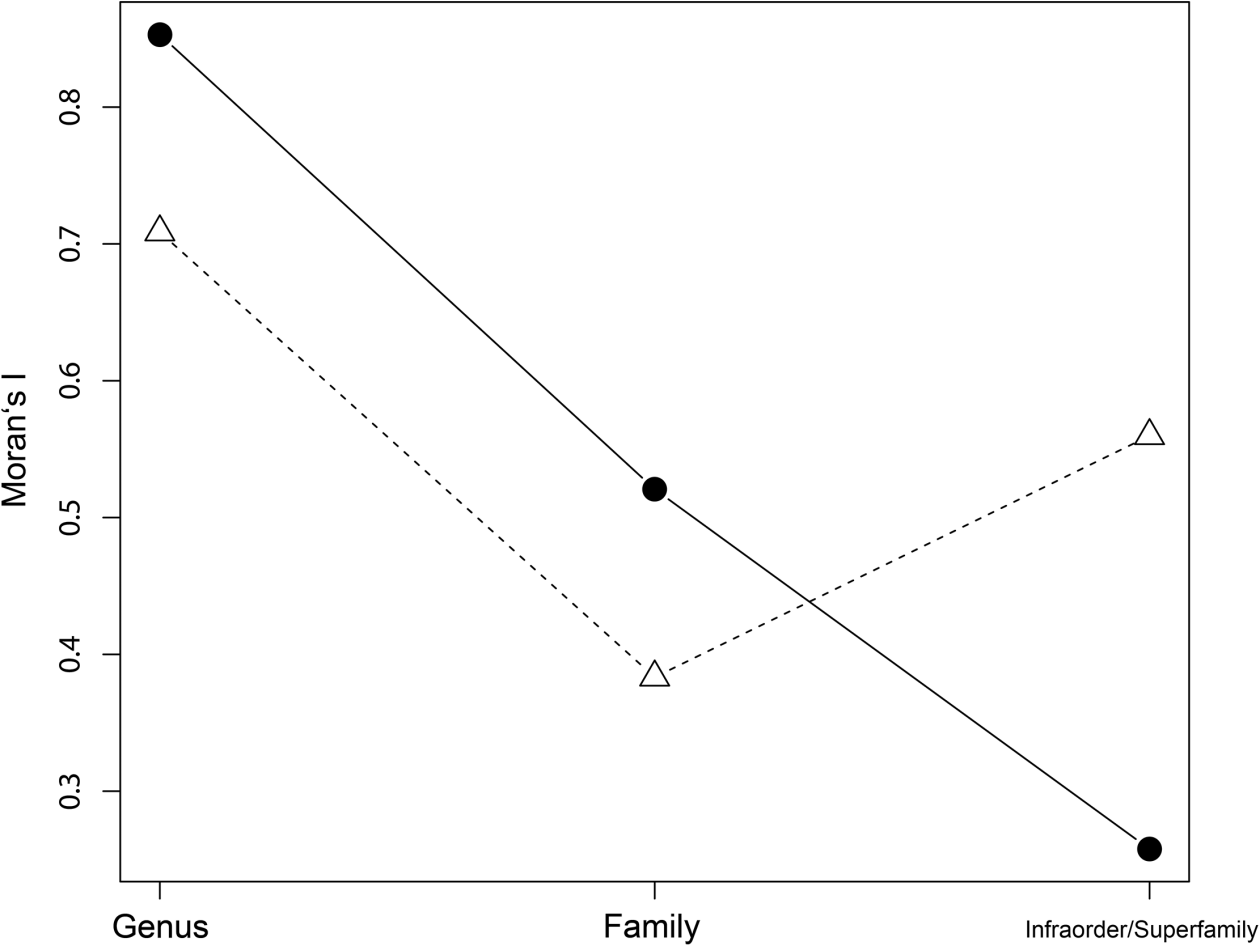
Phylogenetic correlogram showing the strength of the phylogenetic signal in the trait “eggshell type” at different taxonomic levels of the order *Squamata*. The phylogenetic signal was assessed by Moran's I and was calculated for two different datasets (n = 32 and n = 574; see text for details). Unfilled triangle and dashed line: Moran's I values calculated for the 32 squamate species for which we had a complete dataset on life-history traits. Solid circle and solid line: Moran's I values calculated for 574 squamate species, for which the dataset on life-history traits was incomplete. Autocorrelation was significant at all taxonomic levels in both analyses.

The best model in terms of AIC and AICc used a meristic model for trait evolution and the original phylogenetic tree (λ = 1; AIC = 40.76, AICc = 41.65). Assuming no phylogenetic relation (λ = 0) for eggshell types gave the worst fit (meristic: AIC = 67.27, AICc = 67.70), whereas AIC and AICc values were intermediate for λ = 0.5. For the best model based on the original phylogenetic tree and a meristic character states model, the continuous-time Markov model of discrete trait evolution estimated a λ of 1.00 (value close to the bounds of the parameter space) and thus corroborated the previous results (Table 1). The continuous-time Markov models with a fixed λ (= 1) also estimated the transition rates between the eggshell types (Table 1). The meristic trait model predicted a high transition rate from shell-less to parchment-shelled (q_12_ = 0.0036), and a low rate for parchment-shelled to rigid-shelled (q_23_ = 0.0010). For our dataset comprising 32 species the phylogenetic signal of the trait “eggshell type” did not scale with increasing taxonomic levels. The strongest phylogenetic signal on eggshell type was observed at the lowest taxonomic level (genus: Moran's I = 0.71, p < 0.001), but the signal did not clearly diminish with increasing taxonomic levels (Fig 1).

From the genus to the family level there was a decrease in Moran's I (0.38, p < 0.001), whereas it increased again from the family to the infraorder/superfamily level (Moran's I = 0.56, p < 0.001). Contrary, the analysis of 576 squamate species clearly indicated a decrease in Moran’s I from the genus to the infraorder/superfamily level.

#### Life-history traits

Abouheif's test for serial independence (TFSI) revealed different results for life-history traits of the 32 species. It detected significant phylogenetic autocorrelation for birth size (Abouheif's C_mean_ = 0.38, p = 0.001), clutch size (Abouheif's C_mean_ = 0.63, p = 0.001), clutches per year (Abouheif's C_mean_ = 0.40, p = 0.001) and age at female maturity (Abouheif's C_mean_ = 0.18, p = 0.023), but no significant phylogenetic autocorrelation for incubation time (Abouheif's C_mean_ = 0.11, p = 0.126) and maximum longevity (Abouheif's C_mean_ = -0.15, p = 0.855).

### Identification of life-history strategies with PCA and cluster analysis

**Fig 2:**
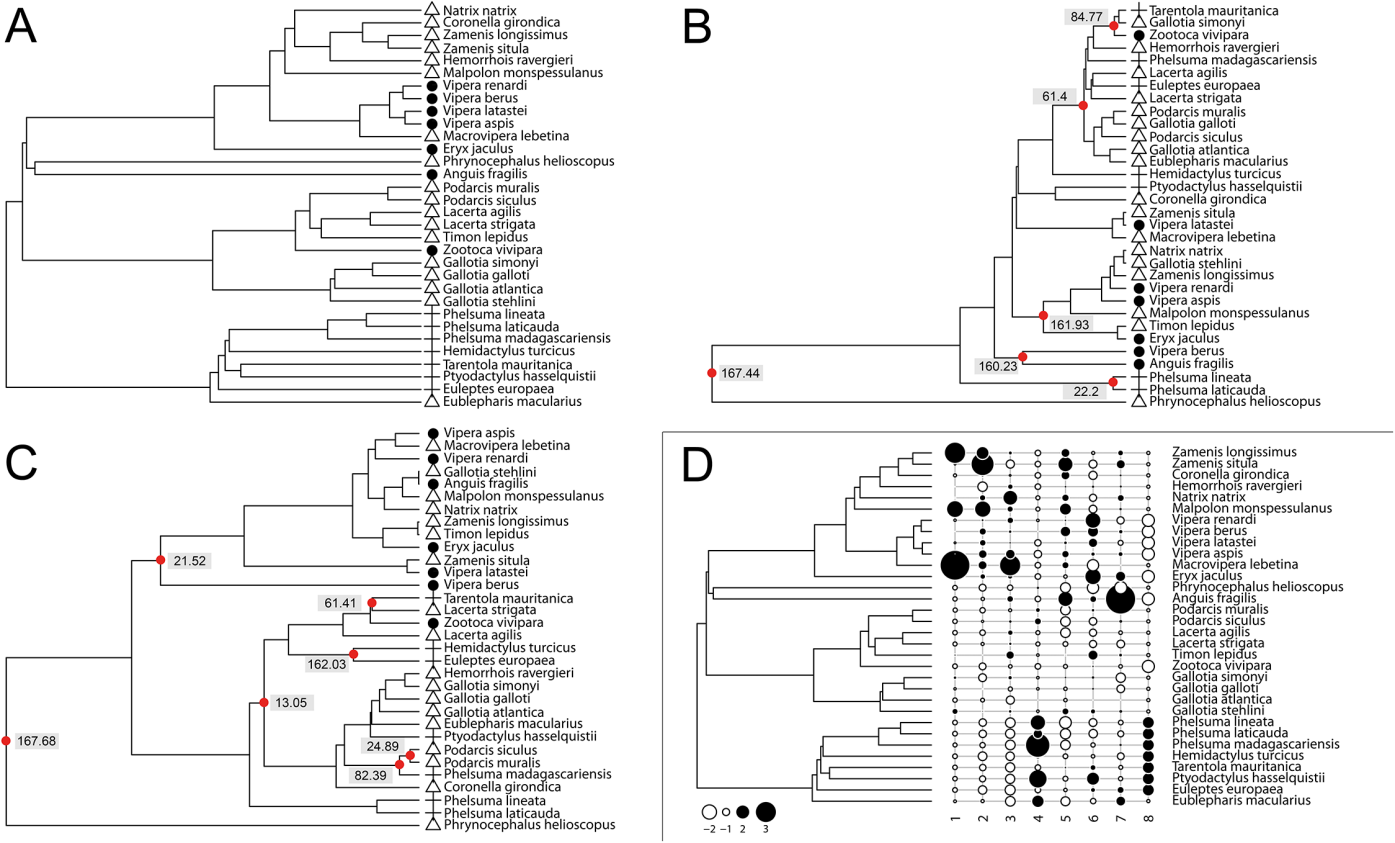
Phenograms of the 32 squamate species based on different analyses and a graphical display of trait values. a) Pruned phylogenetic tree of our squamate dataset derived from a recently published phylogeny (Pyron and Burbrink 2014). b) Phenogram as the result of a cluster analysis based on life-history traits of species. c) Phenogram as the result of a cluster analysis evaluating life-history traits and additionally adult weight of species. d) Graphical display of phylogeny and trait values of species. In a) through c) filled circles = shell-less, open triangles = parchment-shelled, and black crosses = rigid-shelled. In b) and c) red circles indicate internal nodes of trees. The numbers give the differences in branching times as time shifts of internal nodes which are present in both trees. For mean branching times see main text. In d) circle diameters and colours represent values of traits. Horizontal numbers: 1. adult weight, 2. birth size, 3. clutch size, 4. clutches per year, 5. age at female maturity, 6. incubation time, 7. maximum longevity, 8. eggshell type. For trait eggshell: open circles = shell-less, no circles = parchment-shelled, and filled circles = rigid-shelled.

The comparison of the life-history traits based phenogram with the phylogenetic tree (Fig 2A and 2B) indicated differing patterns of relationships for the 32 studied squamate species, and thus their eggshell types (branching times differences: mean = 109.61, median = 122.38, standard deviation σ = 62.17). While species sharing the same eggshell type clearly grouped together in the phylogenetic tree (Fig 2A), there was only a weak association between the life-history strategy of species and eggshell types as shown by the cluster analysis (Fig 2B). A phenogram based on a life-history traits and adult weight of species was more similar to the phylogenetic tree (Fig 2C; branching times differences: mean = 76.14, median = 61.41, σ = 65.32) and species sharing the same eggshell type grouped together (Fig 2C).

**Fig 3:**
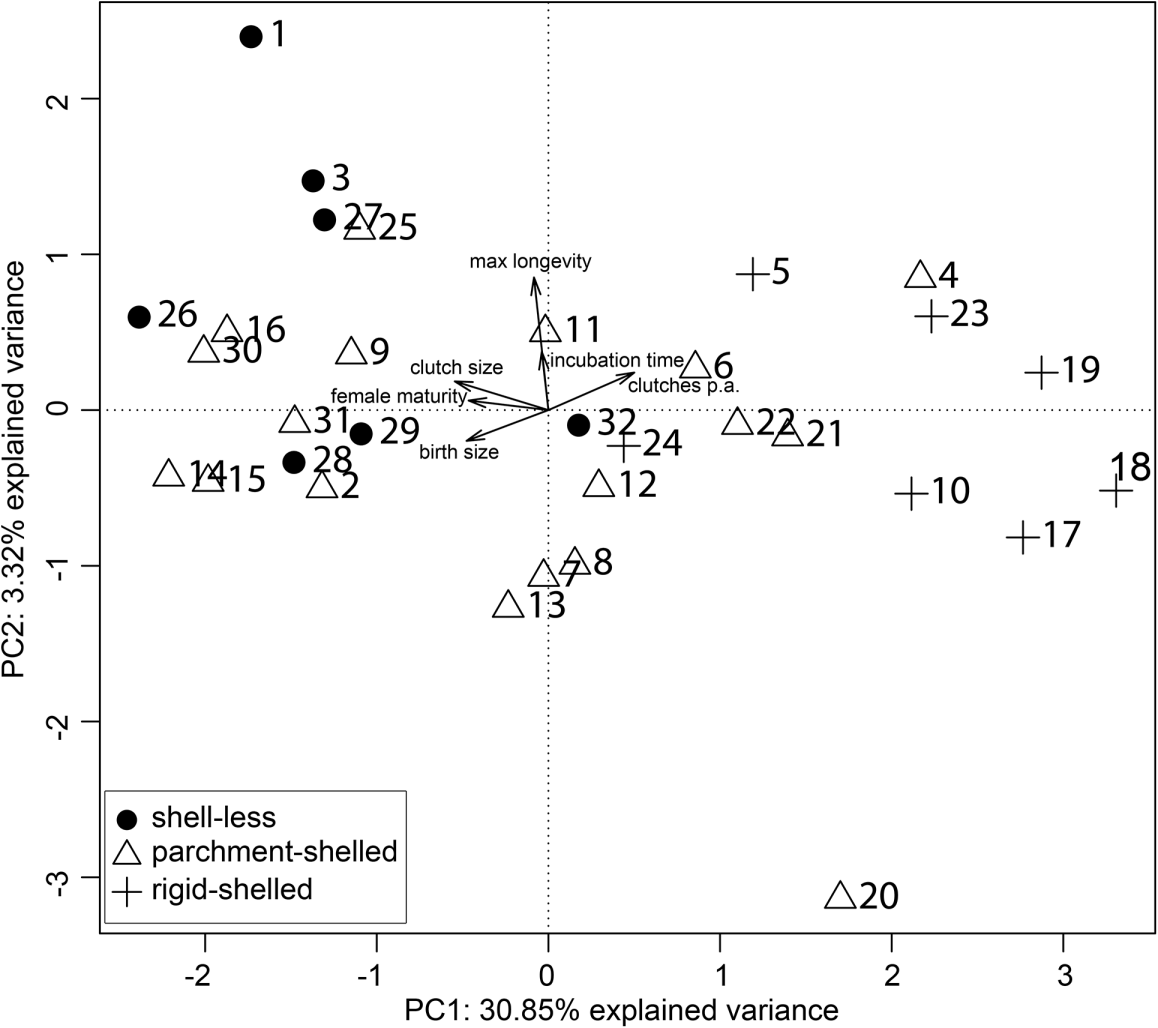
Phylogenetic principal component analysis (pPCA) based on the life history of the studied 32 squamate species with one global principal component (PC1) and one local principal component (PC2). Axes are based on life-history traits of species. Arrows indicate loadings, thus the contribution of life-history traits to PC1 and PC2. The phylogenetic weight matrix was taken from the phylogeny of [51]. In this analysis we did not consider adult weight (see Fig 4). Species numbers: 1. *Anguis fragilis*, 2. *Coronella girondica*, 3. *Eryx jaculus*, 4. *Eublepharis macularius*, 5. *Euleptes europaea*, 6. *Gallotia atlantica*, 7. *Gallotia galloti*, 8. *Gallotia simonyi*, 9. *Gallotia stehlini*, 10. *Hemidactylus turcicus*, 11. *Hemorrhois ravergieri*, 12. *Lacerta agilis*, 13. *Lacerta strigata*, 14. *Macrovipera lebetina*, 15. *Malpolon monspessulanus*, 16. *Natrix natrix*, 17. *Phelsuma laticauda*, 18. *Phelsuma lineata*, 19. *Phelsuma madagascariensis*, 20. *Phrynocephalus helioscopus*, 21. *Podarcis muralis*, 22. *Podarcis siculus*, 23. *Ptyodactylus hasselquistii*, 24. *Tarentola mauritanica*, 25. *Timon lepidus*, 26. *Vipera aspis*, 27. *Vipera berus*, 28. *Vipera latastei*, 29. *Vipera renardi*, 30. *Zamenis longissimus*, 31. *Zamenis situla*, 32. *Zootoca vivipara*. Clutches pa = clutches per annum (per year).

The shell-less snake *Vipera berus* and the shell-less lizard *Anguis fragilis* formed a single cluster, and also the two rigid-shelled species from the gekkotan genus *Phelsuma*. The largest cluster of species that share an eggshell type consisted of five parchment-shelled lizard species (*Eublepharis macularius*, *Gallotia atlantica*, *Gallotia galloti*, *Podarcis muralis*, *Podarcis siculus*). Most of the clusters of the phenogram contained species having two different eggshell types, and one cluster even comprised species from all three eggshell types. The latter group consisted of four lizard species (shell-less: *Zootoca vivipara*, parchment-shelled: *Gallotia simonyi* and rigid-shelled: *Phelsuma madagascariensis*, *Tarentola mauritanica*) and one parchment-shelled snake, the viper *Hemorrhois ravergieri*. In both the phenogram and the phylogenetic tree the parchment-shelled lizard *Phrynocephalus helioscopus* was most distantly related to any of the other species. The phylogenetic principal component analysis (pPCA) indicated a clear clustering of species' life-history strategies based on their eggshell types (Fig 3; Pillai’s trace = 0.48, F(2,29) = 13.21, p < 0.001).

**Fig 4:**
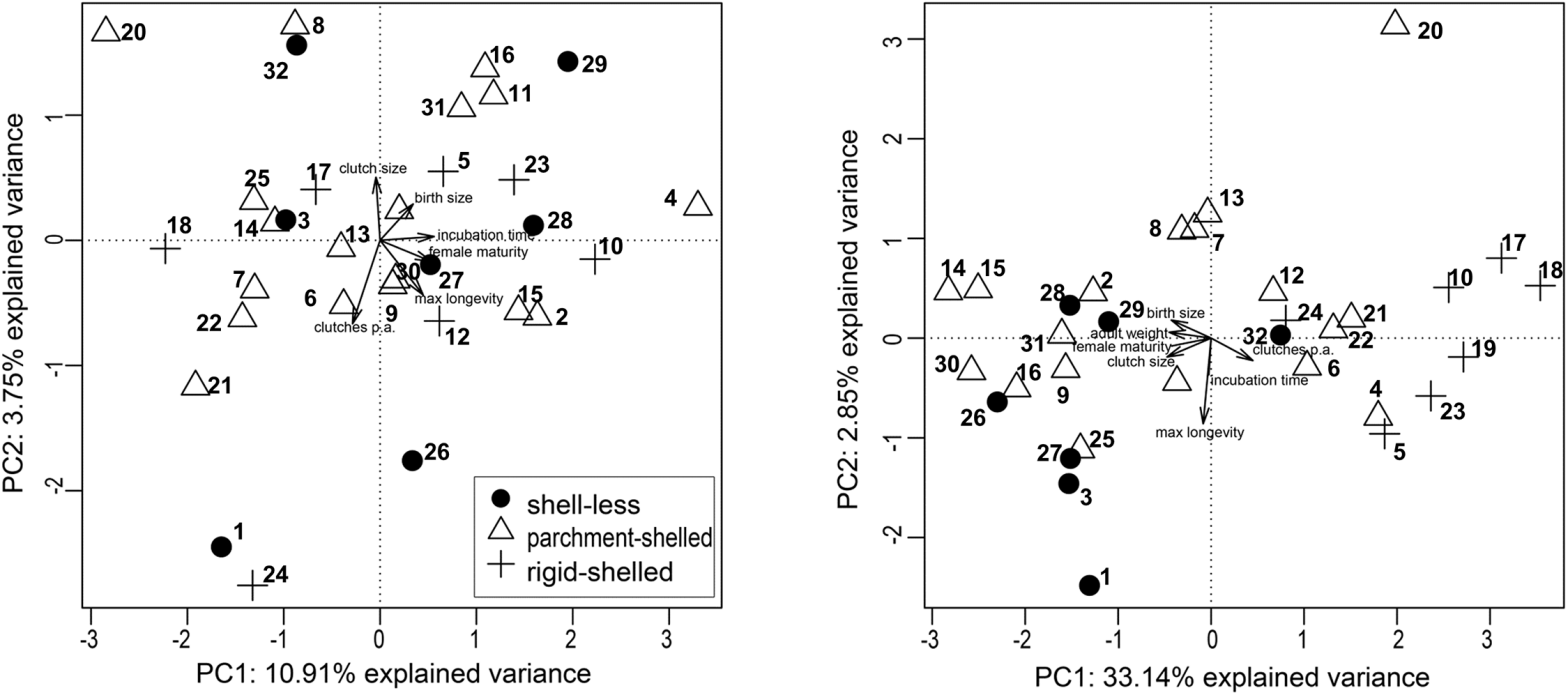
Weight-adjusted principal component analysis (pPCA) based on the life history of the studied 32 squamate species with one global principal component (PC1) and one local principal component (PC2). Axes are based on life-history traits of species. Arrows indicate loadings, thus the contribution of life-history traits to PC1 and PC2. The phylogenetic weight matrix was taken from the phylogeny of [51]). a) In this analysis life-history traits were analysed thereby correcting for adult weight. b) Adult weight was added as further trait to life-history traits. For species numbers please refer to Fig 3. Clutches pa = number of clutches per annum (per year).

All species of the shell-less group, except for *Zootoca vivipara* (only in the two left-hand quadrants), are mapped in the lower right-hand quadrant of the pPCA plot. Species laying rigid-shelled eggs are located in the two right-hand quadrants. The global axis (first principal component, PC1) had a positive eigenvalue of 1.851 and explained 30.85% of the total variance in life-history strategies; the eigenvalue of the local axis PC2 was negative (-0.199) and explained 3.32% of the variance. The trait loadings of PCs suggested different contributions of life-history traits to axes (PC1: birth size = -0.48, clutch size = -0.55, clutches per year = 0.50, age at female maturity = -0.46, incubation time = -0.04, maximum longevity = -0.08; PC2: birth size = -0.20, clutch size = 0.18, clutches per year = 0.24, age at female maturity = 0.06, incubation time = 0.37, maximum longevity = 0.85). PC1 was strongest influenced by the traits age at female maturity, birth size, clutches per year and clutch size, whereas PC2 by incubation time and maximum longevity. The adult weight-adjusted pPCA did not reveal any association of eggshell types and life-history strategies (Fig 4A; Pillai’s trace < 0.01, F(2,29) = 0.03, p = 0.97).

Species of any eggshell type were found in all quadrants of the adult weight-adjusted pPCA plot. Further, the trait loadings indicated differing contributions of traits to the two principal components (PC1: birth size = 0.34, clutch size = -0.04, clutches per year = -0.29, age at female maturity = 0.53, incubation time = 0.56, maximum longevity = 0.45; PC2: birth size = 0.29, clutch size = 0.50, clutches per year = -0.67, age at female maturity = -0.17, incubation time = 0.03, maximum longevity = -0.44). Contrary to the non-weight-adjusted pPCA, the main contributors to PC1 were age at female maturity, incubation time and maximum longevity, and for PC2 clutch size, clutches per year and maximum longevity. The eigenvalues of both axes were low compared to the non-weight-adjusted pPCA with 0.655 for PC1 and -0.225 for the PC2 and the amount of variability explained by the two PCs was much lower (PC1: explained variance = 10.91%; PC2: 3.75%). However, a non-weight-adjusted pPCA considering adult weight as a further trait (Fig 4B) besides the life-history traits of species retained the association of eggshell types and life-history strategies and led to a PC1 with a higher degree of variance explained (33.14%; Pillai’s trace = 0.44, F(2,29) = 11.24, p < 0.001).

## Discussion

### Phylogenetic signals of eggshell types and life-history traits

The hypothesis that ecological and phylogenetic similarity between species are linked [75–77] was one of the main drivers for this study. Blomberg et al. [78] were already able to show for phylogenetic trees with 20 or more species that for a broad range of living organisms 92% of the analysed traits exhibited a significant phylogenetic signal. We asked in our study whether related squamate species share the same eggshell type. Our investigation clearly showed that the discrete trait “eggshell type” has a strong phylogenetic signal (Table 1). It is a meristic trait, which evolves under Brownian motion (estimated lambda λ = 1.00). We further showed, that the phylogenetic signal vanishes with increasing distances from the root to the tips of the phylogenetic tree (λ < 1), indicating a location of the signal at a low taxonomic level (Table 1). This is consistent with the result of the phylogenetic correlogram (Fig 1), which has the advantage of not explicitly assuming an evolutionary model [79]. The noticeably higher Moran’s I value for the infraorder/superfamily level in the correlogram seems to be an artefact of the small dataset (n = 32), because the same analysis applied to a larger dataset of 574 squamate species showed a steady decline with higher taxonomic levels for eggshell type (Fig 1). If we interpret the discrete trait “eggshell type” as a morphological trait (e.g. shell thickness or shell strength), we can follow the suggestion from Wiens and Slingluff [80] and treat the eggshell type as a continuous and quantitative character. In this case, a likelihood model of continuous character evolution [61,76,78] confirmed the results of the discrete trait evolution model for eggshell types (AIC = 34.60, AICc = 35.49, λ = 1, close to bounds of the parameter space). Thus, regardless of whether the trait”eggshell type” is treated as a discrete or continuous character, it has a strong phylogenetic signal.

To assess the evolutionary distances between eggshell types, we estimated the transition rates between eggshell types assuming the meristic model, which got the highest statistical support (Table 1). It predicts a higher likelihood for a transition between the shell-less type and the parchment-shelled type than for a transition between the parchment-shelled type and rigid-shelled type. This indicates that there is less evolutionary distance between the shell-less and the parchment-shelled type, than between the parchment-shelled and rigid-shelled type. This result is unexpected, as the transition from parchment-shelled to shell-less, thus oviparity to viviparity, has involved more physiological changes like egg-retention or formation of placenta-like structures [11,81–83] than the transition from parchment-shelled to rigid-shelled eggs. The emergence of the rigid-eggshell type was a unique evolutionary event within the squamates, rigid eggshells only exist in some gekkotan families [3,17]. It has been argued that the rigid eggshell is only a special variant of the parchment-shelled type, because after oviposition the eggs are still soft and during the subsequent hardening process the eggs are transformed within about one hour into rigid-shelled eggs [3,36–38]. We also explored whether a phylogenetic signal is seen in the life-history traits to understand their possible association. This analysis revealed a dichotomy for species’ life-history traits: the phylogenetically related traits (birth size, clutch size, clutches per year and age at female maturity) and the traits lacking such a relation (incubation time and maximum longevity). Few studies on the influence of the shared evolutionary history on the life-history traits including those investigated by us exist for different squamate taxa (summarized in Blomberg et al. [78]). For lizards, especially traits concerning body size do in general possess a phylogenetic signal [20,84], e.g. body mass for members of the genus *Anolis* [85] and SVL or tail length for the family *Anguidae* [80]. But exceptions have also been reported, such as the iguana family *Phrynosomatidae*, where body size is not influenced by a shared evolutionary history [86]. For the life-history traits of lizards, studied by Clobert et al. [84], a significant phylogenetic signal existed for the traits age at maturity, clutch size and number of clutches per year after correcting traits for snout-vent-length (SVL). When we corrected the traits in our dataset for adult mass, this resulted in a complete loss of phylogenetic relatedness for all traits except for incubation time, which now showed a significant phylogenetic signal (Abouheif's C_mean_ = 0.23, p = 0.03). However, we think that these results provide further evidence that not all traits correlate to their phylogenetic history [68,78].

### Relation between eggshell types and life-history traits and the influence of body mass

We tested the hypothesis, that species which share the same eggshell type possess similar characteristics of life-history traits. As the trait “eggshell type” has a strong phylogenetic signal, it can be seen as a substitute for phylogeny (see discussion about “Phylogenetic signal of eggshell and life-history traits”). Consequently, phylogenetically related species should also have the same eggshell type. This expectation is supported by the phylogenetic tree [60], which shows a grouping of phylogenetically related species sharing the same eggshell type (Fig 2B). If life-history strategies are associated with particular eggshell types, the phenogram derived from cluster analysis of life-history traits should resemble the phylogenetic tree. Interestingly, this phenogram and the phylogenetic tree differed (Fig 2A and 2B). There was no clear consensus on the positions of squamate species in both, and thus of eggshell types. In the cluster analysis based phenogram there were mostly groups of species which represented two eggshell types. Only the combinations shell-less/parchment-shelled and parchment-shelled/rigid-shelled could be found, which supports the finding that eggshell type is a meristic character. However, there was one group (*Gallotia simonyi*, *Hemorrhois ravergieri*, *Tarentola mauritanica*, *Zootoca vivipara*), which comprised members of all three eggshell types. Here, the differences between the phenogram from cluster analysis and the phylogenetic tree were strongest. Especially the position of the only snake of this group, *H*. *ravergieri*, differed strongly between the phenogram and the phylogenetic tree (Fig 2A and 2B). The reason for this could be the exceptionally low age at female maturity and the small birth size of *Hemorrhois ravergieri* compared to other snakes. If we included adult weight as a further parameter in our cluster analysis, *H*. *ravergieri* was placed close to parchment-shelled species with a more similar adult weight (Fig 2C). This also happened for *Gallotia simonyi*, the second species of this group (Fig 2C). The third species of this group, the rigid-shelled *Tarentola mauritanica*, clustered together with species of different eggshell types but similar adult weights (Fig 2C). Thus, adult weight seems to have a greater effect on the positioning of the squamate species within the life-history trait based phenogram than the other life-history traits studied. The fourth species of this cluster, the shell-less species *Zootoca vivipara*, is extremely widespread. This reptile has the most northern distribution with presences up to the 70^th^ parallel of latitude of all squamate species. Individuals of northern populations can be viviparous (shell-less eggs), as assumed for this species in our analyses, whereas those of extreme southwestern populations lay parchment-shelled eggs [55,56,66,87]. Life-history traits also show large variability due to local adaption and reflect the broad range of environmental conditions that are found within the distribution range of *Z*. *vivipara*. This explains the considerable distance of *Z*. *vivipara* to the other shell-less species in the cluster analysis based phenogram. The parchment-shelled agama *Phrynocephalus helioscopus* has the highest distance to each of the other species in this phenogram (Fig 2B and 2C). Its considerable shorter average lifespan of 2.5 years compared to that of all other species (Darewskij, cited in Böhme, [24]) have added to these large distances. A low lifespan is also seen in other species of the genus *Phrynocephalus*. For example, *P*. *guttatus* individuals can live for 1–2 years, and in a *P*. *mystaceus* population only few animals lived up to 6 years [24]. In conclusion, the order of species in the life-history traits based phenogram matched only to a low degree their order in the phylogenetic tree, and thus of the eggshell types, but the degree of consensus between both increased if adult weight was additionally included in the analysis. We thus conclude that life-history traits reflect only to a certain amount the shared evolutionary history of squamate species. This is consistent with our results on a phylogenetic signal in life-history traits (see discussion about “Phylogenetic signal of eggshell types and life-history traits”). Consequently, to derive to what extent life-history traits are related to particular eggshell types and vice versa, we removed the phylogenetic signal in the pPCA, and also the effect of body size. Additionally, the findings from the trait-based phenogram necessitated taking care of the effect of body size as well. After removal of a phylogenetic signal by utilizing a phylogenetic principal component analysis (pPCA), we could identify distinct life-history strategies for all of the three eggshell types (Fig 3). The pPCA revealed a strongly explanatory global axis (30.8% explained variance) associated with large positive Moran’s I values and a weak explanatory local axis (4.4% explained variance) associated with large negative Moran’s I values. Jombart et al. [88], however, recommended an interpretation of the local axis even when the corresponding eigenvalue is small in comparison to that of the global axis, if it is biologically informative. Following the suggestion of that paper, the local axis predicts that the shell-less type corresponds with a lifestyle characterized by late sexual maturity of females, production of few, large clutches and offspring with a large birth size. Shell-less species also have a considerable higher life expectancy, while incubation time (or more precisely gestation time) is prolonged compared to rigid-shelled species. For the reasons explained above, the life-history traits of *Z*. *vivipara* did not match this shell-less strategy. Rigid-shelled species show early sexual maturity, a production of multiple, small clutches and have offspring of a small birth size. There were no differences in terms of lifespan or incubation time compared to the shell-less or parchment-shelled type. Parchment-shelled species were the most diverse group with respect to life-history strategies. They either covered the strategy of the shell-less and rigid-shelled species, or had an intermediate strategy with respect to the age at female maturity, clutch size, frequency of clutches per year and birth size. Our results on life-history strategies in squamates did not completely corroborate the results on gekkotan species [17]. While consistent with the findings of Pike et al. [17] the offspring of rigid-shelled squamates had smaller birth sizes than of parchment-shelled squamates (rigid-shelled: mean 4.31, parchment-shelled: mean 12.77; t = 4.01; p < 0.001), contrary to these authors incubation time did not differ between these two eggshell types (rigid-shelled: mean 67.29; parchment-shelled: mean 58.28; t = -1.10; p = 0.30).

**Fig 5:**
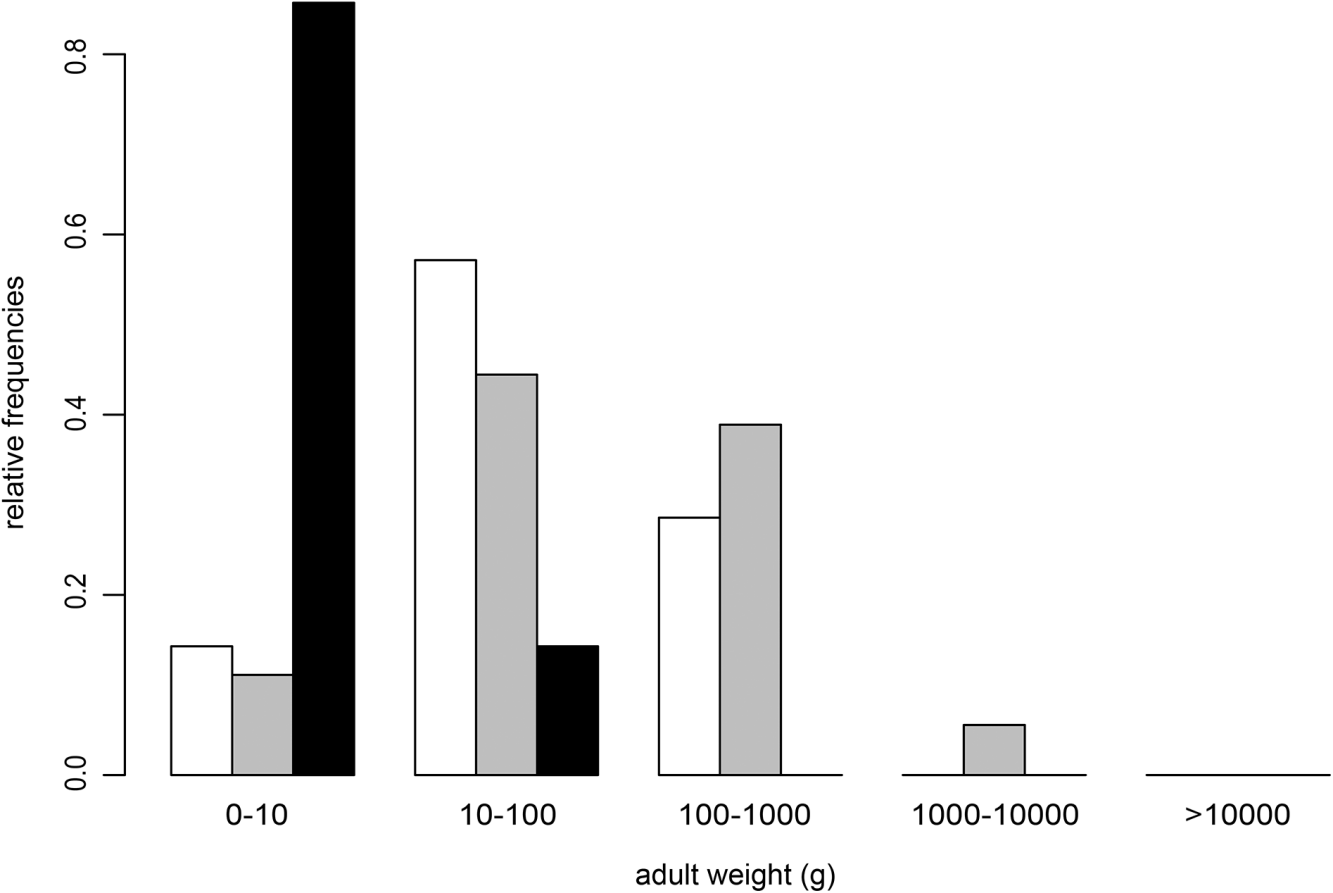
Relative frequencies of adult weights (in grams) of the analysed 32 squamate species presented for each of the three eggshell types. White = viviparous, grey = parchment-shelled eggs, black = rigid-shelled eggs.

Analyses of reproductive strategies seen in different squamate groups have been carried out by several authors [14–16,89–92]. Tinkle et al. [15] proposed different reproduction strategies for oviparous and viviparous lizards. Oviparous lizards mature early and produce clutches frequently, the reproduction strategy that is seen in the rigid-shelled squamates [15]. Viviparous lizards mature late and produce few, small or large clutches [15], which is consistent with our characterisation of the shell-less squamates, although the shell-less species in our dataset were mostly snakes and not lizards. Tinkle et al. [15] also described two intermediary reproduction strategies, which resemble the strategy of our parchment-shelled species. One strategy combines early reproduction and multiple, small clutches and the other a delayed reproduction with one large clutch per year [15]. Dunham et al. [16] validated the results of Tinkle et al. [15] for lizards and repeated their analyses for snakes. They identified three reproductive strategies in snakes: single-brooded, oviparous species, as well as annually-breeding and biennially-breeding viviparous species [16]. The biennially-breeding viviparous species are larger, have larger clutches and a more delayed maturity than the annually-breeding viviparous species [16]. As all shell-less species in our study (except for *Z*. *vivipara*) are snakes or snake-like (*Anguis fragilis*), this dichotomy in life-history strategies of viviparous snakes partly corroborates our unclear results on the life-history strategy of shell-less species. Stearns [93] already analysed the effects of body size and phylogeny on patterns of covariation in life-history traits of lizards and snakes. He recognized that, although these patterns are influenced by phylogeny, the main reason for the patterns of covariation is the correlation of the life-history traits with the average adult female length, and thus body size [93]. Dunham and Miles [89] questioned the results of Stearns [93]. After removing size effects from a corrected version of Stearns’ dataset, they demonstrated a significant impact of phylogeny on the covariation in life-history traits. Dunham et al. [16] argued that similarities between life-history strategies of squamate species most likely result from physiological constraints that are linked to the mode of reproduction and to breeding frequency [16]. In our study, the original grouping of eggshell types in the pPCA diagram almost completely vanished when life-history traits were corrected for adult weight and their residuals were used (Fig 4A). This observation points to a strong size effect on life-history traits as suggested by Stearns [93]. When adult weight was included as a further life-history trait in the pPCA (Fig 4B), the original grouping of eggshell types was recovered, which points to a stronger effect of phylogeny on life-history strategies and thus corroborates Dunham and Miles [89]. Differences in adult body sizes between parchment-shelled and rigid-shelled gekkotan species were also observed by Pike et al. [17]. In our study on squamate species the mean adult weight also differed between the eggshell types (Kruskal-Wallis test χ^2^ = 11.94, df = 2, p-value < 0.01, Fig 5).

Overall, we conclude from our analyses that a distinct eggshell type is correlated with a particular body size, which in turn is associated with a distinct life-history strategy.

However, the squamate dataset analysed by us was small, because only for 32 species all studied traits were available, and the phylogeny of squamates as well as the geographic distribution of the eggshell types could have been insufficiently covered. We therefore repeated the pPCAs with a dataset comprising 300 worldwide distributed squamates and only 5 life-history traits for each species from a recent publication [47]. This dataset lacks direct measurements of body mass of species (g), and estimated species’ body mass from length-weight allometries (snout-vent-length or total length of animals). We think that this approach is problematic as large differences between the estimated and the real body mass can result (see S2 Table). Nevertheless, the results of a pPCA for a dataset which is an order of magnitude larger (300 species) and covers fewer life-history traits, resembled the results obtained for our 32 studied squamates (see S1 Fig). The rigid-shelled and the shell-less species were clearly separated in the pPCA plot, while the parchment-shelled species were found in each of the quadrants. When we corrected for body mass in this pPCA again the explained variance decreased significantly and the previously found pattern disappeared (see S2 Fig).

Our results indicate a high impact of body mass on life-styles associated with egg shells. However, metabolic rate influences all levels of biological organization including life-histories of species [94]. Since metabolic rate and body mass are linked (for squamates see [95–97]), an analysis disentangling the effects of body mass and metabolic rate on the association of eggshell types and life-histories of squamates would be very intriguing, but the inclusion of metabolic rate in this study would have presumably further reduced our dataset, because data on metabolic rates are insufficiently available for the species under study.

## Supporting Information

S1 Table Trait values and literature sources of examined 32 squamates species. (PDF)

S2 Table Comparison between measurements of adult weight (g) and weights estimated from length-weight allometries for the examined 32 squamata species. (PDF)

S3 Table Results of multiple, phylogenetic regression analyses (PGLS) with maximum altitude (m) and adult weight (g) as predictors of life-history traits. (PDF)

S1 Fig Phylogenetic principal component analysis (pPCA) with two global principal components (PC1/PC2) for 5 life-history traits of 300 squamate species. (PDF)

S2 Fig Weight-adjusted principal component analysis (pPCA) with two global principal components (PC1/PC2) for life-history traits of 300 squamate species. (PDF)
