# Supplementary material for: Eggshell Types and Their Evolutionary Correlation with Life-History Strategies in Squamates
Source: PLoS One. 2015 Sep 22;10(9):e0138785. doi: 10.1371/journal.pone.0138785 (PMC4579135; doi:10.1371/journal.pone.0138785)
Supplement: S1 Table — (PDF) [file pone.0138785.s001.pdf]

S1 Table Trait values and literature sources of examined 32 squamata species. Clutches p.a. = number of clutches per annum (per year); eggshell type classification: 1 = shell-less, 2 = parchment-shelled, 3 = rigid-shelled. Literature numbers: 1. Böhme (1981), 2. Deckert et al. (1981), 3. Böhme (1984), 4. Böhme (1985), 5. Böhme (1988), 6. Kabisch (1990), 7. Henkel and Schmidt (1991), 8. Rogner (1992), 9. Böhme (1993), 10. Rösler (1995), 11. Böhme (1999), 12. Foufopoulos et al. (1999), 13. Gasso (2003), 14. Heygen (2004), 15. Joger and Stümpel (2005), 16. Agasyan et al. (2009), 17. Green et al. (2009), 18. Wise (2009), 19. Meiri (2010), 20. Delaugerre et al. (2011), 20. Raxworthy et al. (2011), 21. Vences (2011), 22. Feldman and Meiri (2013), 23. Tacutu et al. (2013), 24. Yu et al. (2014). For a more detailed description of the literature see main text.

	Species	Adult weight (g)	Birth size (cm)	Clutch size	Clutches p.a.	Female maturity (days)	Incubation time (days)	Max. longevity (years)	Eggshell type	Max. altitude (m)	Literature
**1**	*Anguis fragilis*	14	8	12.25	1	1825	84	44.33	1	2400	1,2,6,17,19, 24
**2**	*Coronella girondica*	50.22	15.02	6.86	1	1460	47.5	14	2	3200	9
**3**	*Eryx jaculus*	134.5	16.75	11.33	1	730	107	24.7	1	1700	9,22,23
**4**	*Eublepharis macularius*	59.7	8.18	1.75	4	400	54.25	24.25	2	2500	2,7,8,10,17, 18,19,24
**5**	*Euleptes europaea*	1.5	3	1.5	1	730	79.5	21.5	3	450	1,2,7,10
**6**	*Gallotia atlantica*	12.8	7.56	2.68	1.95	730	68.25	15	2	580	2,5,19
**7**	*Gallotia galloti*	72.75	12.33	5.24	1.5	730	65.86	7.03	2	2000	2,5,19,24
**8**	*Gallotia simonyi*	252.31	3.63	10.8	1.5	913	64.5	5.4	2	500	2,5,24
**9**	*Gallotia stehlini*	326.5	13.23	10.33	1.5	1346.5	80	13	2	1950	5,19,24
**10**	*Hemidactylus turcicus*	2.8	2.8	2.5	2.88	650	58.75	7.9	3	1100	1,7,9,19,24
**11**	*Hemorrhois ravergieri*	157.75	2.05	11.5	1	913	65	13.8	2	3300	9,23
**12**	*Lacerta agilis*	8.3	5.89	11.5	1.25	365	50	11	2	2400	3,6,19
**13**	*Lacerta strigata*	20.6	8.25	10.17	1.25	645	50	7	2	2000	3,19
**14**	*Macrovipera lebetina*	1037.83	20.04	21.67	1	1350	40	15.15	2	2500	15,24
**15**	*Malpolon monspessulanus*	649.92	27.26	11.95	1.25	1642.5	48.5	13.5	2	2000	11
**16**	*Natrix natrix*	190.16	17.35	17.51	1.25	1369	50	21.42	2	2300	11,24
**17**	*Phelsuma laticauda*	2.9	4	2	3.5	330	46.88	8.7	3	300	7,8,10,14,1 9,24
**18**	*Phelsuma lineata*	3.8	4	1.75	4.5	240	48.25	10	3	1350	7,8,10,19,2 3,24
**19**	*Phelsuma*	20.7	6.07	1.75	6	367.5	56.94	13.6	3	1000	7,8,10,19,2

	Species	Adult weight (g)	Birth size (cm)	Clutch size	Clutches p.a.	Female maturity (days)	Incubation time (days)	Max. longevity (years)	Eggshell type	Max. altitude (m)	Literature
	madagascariensis										1,24
**20**	*Phrynocephalus helioscopus*	6.9	4.55	4.5	1.75	345	40	2.5	2	1000	1,6,19
**21**	*Podarcis muralis*	15.18	6.05	6	2.75	365	76.25	8.5	2	2700	4,19,24
**22**	*Podarcis siculus*	10.75	9.13	7.17	3.1	365	49.11	13	2	2000	4,19
**23**	*Ptyodactylus hasselquistii*	9.3	5.3	2	5	638.75	99.25	10.45	3	1800	10,19,24
**24**	*Tarentola mauritanica*	7.3	5	2	1	1095	81.5	10.7	3	1283	1,7,8,10,19, 24
**25**	*Timon lepidus*	213.28	11.76	13.38	1.5	1026	91.75	18.5	2	2100	2,3,17,19,2 4
**26**	*Vipera aspis*	74.1	19.7	14.16	0.69	1369	78	19.5	1	3000	15,22,24
**27**	*Vipera berus*	153.15	18.3	10.33	2	1551.5	94.33	19	1	3000	2,6,15,17,2 4
**28**	*Vipera latastei*	87	17.67	7.91	0.88	1156	90	9.7	1	3030	15,22,24
**29**	*Vipera renardi*	71.8	14.2	12.17	1.5	1095	105.6	7.5	1	2500	15
**30**	*Zamenis longissimus*	783.33	23.85	10.6	1	1460	60	20.33	2	1700	9,24
**31**	*Zamenis situla*	95.5	33.83	2.67	1	1824.5	48	23.5	2	1260	9,24
**32**	*Zootoca vivipara*	3.9	4.53	5.67	1	6.95	74	11	1	2400	3,17,19,23
