# Supplementary material for: Eggshell Types and Their Evolutionary Correlation with Life-History Strategies in Squamates
Source: PLoS One. 2015 Sep 22;10(9):e0138785. doi: 10.1371/journal.pone.0138785 (PMC4579135; doi:10.1371/journal.pone.0138785)
Supplement: S2 Table — (PDF) [file pone.0138785.s002.pdf]

S2 Table. Comparison between measurements of adult weight (g) and weights estimated from length-weight allometries for the examined 32 squamate species. The devation (in %) between real and estimated body mass ranges from 2.80% and 91.80% (mean = 39.81%, SD = 24.92; median = 33.55%). All calculations were based on length-weight allometries established either by Meiri (2010) or Feldman and Meiri (2013). For the lizards we used the PGLM (phylogenetic general linear model) equation based on snout-vent-length (SVL): $\log_{10}(\text{mass}) = (3.053 \times (\log_{10}\text{SVL})) - 4.727$. Only for the species *Anguis fragilis*, we used the PGLM equation for legless lizards: $\log_{10}(\text{mass}) = (2.232 \times (\log_{10}\text{SVL})) - 4.499$. For body mass calculations in snakes we used the equation based on total length (TL): $\log_{10}(\text{mass}) = (2.443 \times ((\log_{10}\text{TL})) - 4.989$.

	Species	Adult weight (g)	Estimated adult weight (g)	Deviation (%)
1	*Anguis fragilis*	14.00	6.28	55.18
2	*Coronella girondica*	50.22	79.65	58.61
3	*Eryx jaculus*	134.50	73.30	45.51
4	*Eublepharis macularius*	59.70	47.36	20.67
5	*Euleptes europaea*	1.50	1.35	9.96
6	*Gallotia atlantica*	12.80	9.34	27.02
7	*Gallotia galloti*	72.75	39.81	45.27
8	*Gallotia simonyi*	252.31	164.71	34.72
9	*Gallotia stehlini*	326.50	275.04	15.76
10	*Hemidactylus turcicus*	2.80	3.55	26.80
11	*Hemorrhois ravergieri*	157.75	299.27	89.71
12	*Lacerta agilis*	8.30	0.88	12.52
13	*Lacerta strigata*	20.60	26.68	29.52
14	*Macrovipera lebetina*	1037.83	387.61	62.65
15	*Malpolon monspessulanus*	649.92	271.39	58.24
16	*Natrix natrix*	190.16	299.12	57.30
17	*Phelsuma laticauda*	2.90	3.46	19.14
18	*Phelsuma lineata*	3.80	4.13	8.73
19	*Phelsuma madagascariensis*	20.70	20.12	2.81
20	*Phrynocephalus helioscopus*	6.90	4.08	40.93
21	*Podarcis muralis*	15.18	6.10	59.79
22	*Podarcis siculus*	10.75	12.42	15.58
23	*Ptyodactylus hasselquistii*	9.30	12.11	30.21
24	*Tarentola mauritanica*	7.30	11.42	56.42
25	*Timon lepidus*	213.28	144.23	32.38
26	*Vipera aspis*	74.10	129.96	75.38
27	*Vipera berus*	153.15	122.37	20.10
28	*Vipera latastei*	87.00	38.27	56.01
29	*Vipera renardi*	71.80	46.21	22.74
30	*Zamenis longissimus*	783.33	856.11	9.29
31	*Zamenis situla*	95.50	183.17	91.80
32	*Zootoca vivipara*	3.90	7.14	83.17
