# Supplementary material for: Eggshell Types and Their Evolutionary Correlation with Life-History Strategies in Squamates
Source: PLoS One. 2015 Sep 22;10(9):e0138785. doi: 10.1371/journal.pone.0138785 (PMC4579135; doi:10.1371/journal.pone.0138785)
Supplement: S3 Table — (PDF) [file pone.0138785.s003.pdf]

S3 Table. Results of multiple, phylogenetic regression analyses (PGLS) with maximum altitude (m) and adult weight (g) as predictors of life-history traits. Phylogeny from Pyron & Burbrink (2013), Pyron et al. (2014). Degrees of freedom = 32. Data was on logarithmic scale (log_{10}).

Trait	Intercept	p-value	Max. altitude	P-value	Adult weight	P-value	λ	AIC
**birth size**	-0.139±0.528	0.795	0.211±0.173	0.234	0.253±0.059	<0.001	0.00	14.29
**clutch size**	-0.019±0.500	0.970	0.145±0.156	0.360	0.212±0.061	0.002	0.76	6.37
**clutches p.a.**	-0.046±0.474	0.923	0.065±0.145	0.658	0.033±0.059	0.581	0.94	5.25
**female maturity**	3.017±0.384	<0.001	-0.086±0.116	0.465	0.099±0.048	0.046	0.98	-6.33
**incubation time**	1.536±0.268	<0.001	0.090±0.091	0.330	-0.011±0.026	0.683	-0.13	-20.94
**max. longevity**	0.760±0.546	0.175	0.081±0.171	0.638	0.059±0.067	0.388	0.75	11.46
