# Supplementary material for: Eggshell Types and Their Evolutionary Correlation with Life-History Strategies in Squamates
Source: PLoS One. 2015 Sep 22;10(9):e0138785. doi: 10.1371/journal.pone.0138785 (PMC4579135; doi:10.1371/journal.pone.0138785)
Supplement: S1 Fig — (PDF) [file pone.0138785.s004.pdf]

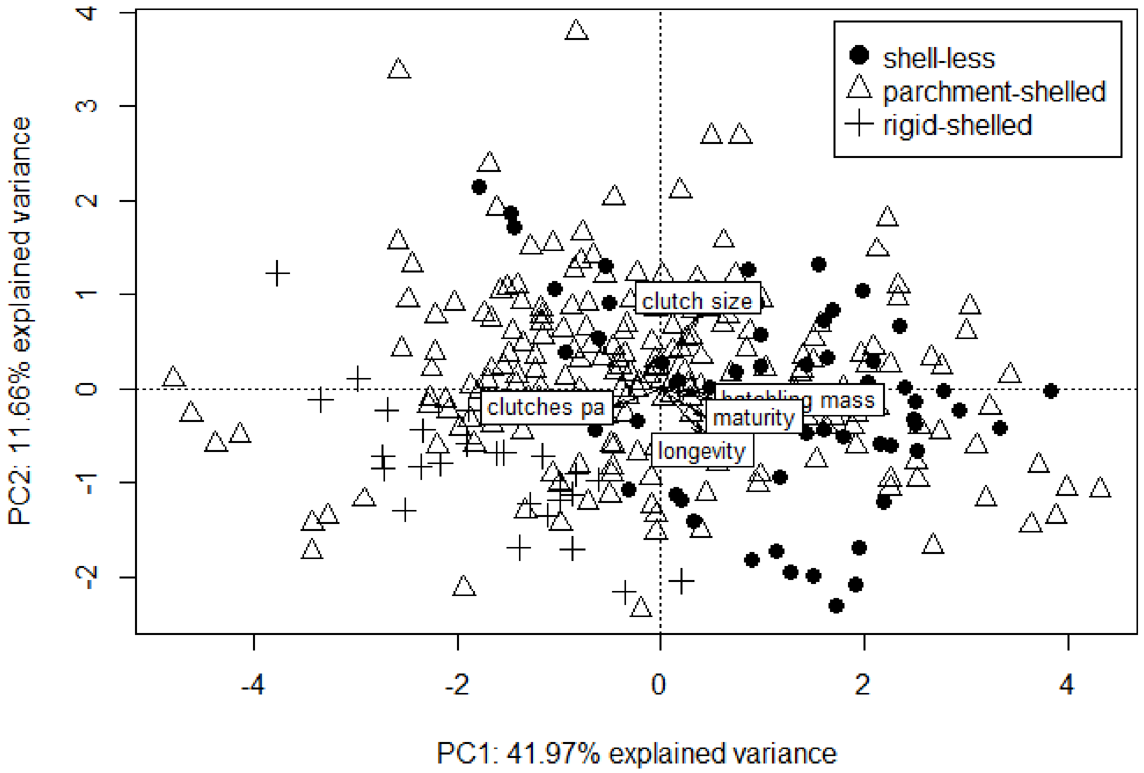

S1 Fig. Phylogenetic principal component analysis (pPCA) with two global principal components (PC1/PC2) for 5 life-history traits of 300 squamate species. Dataset is taken from Scharf et al. (2014). Instead of direct measurements of adult weight (g), Scharf et al. (2014) used length-weight allometries to estimate the body mass of squamate species from snout-vent-length (SVL) or total length (TL). Axes are based on life-history traits of species. Arrows indicate loadings, thus the contribution of life-history traits to PC1 and PC2. The phylogenetic weight matrix was taken from the phylogeny of Pyron et al. (2013). Loadings of PC1: hatchling mass = 0.52, clutches pa = -0.47, clutch size = 0.37, maturity = 0.45, longevity = 0.41; loadings of PC2: hatchling mass = -0.12, clutches pa = -0.20, clutch size = 0.79, maturity = -0.30, longevity = -0.48.
