# Supplementary material for: Eggshell Types and Their Evolutionary Correlation with Life-History Strategies in Squamates
Source: PLoS One. 2015 Sep 22;10(9):e0138785. doi: 10.1371/journal.pone.0138785 (PMC4579135; doi:10.1371/journal.pone.0138785)
Supplement: S2 Fig — (PDF) [file pone.0138785.s005.pdf]

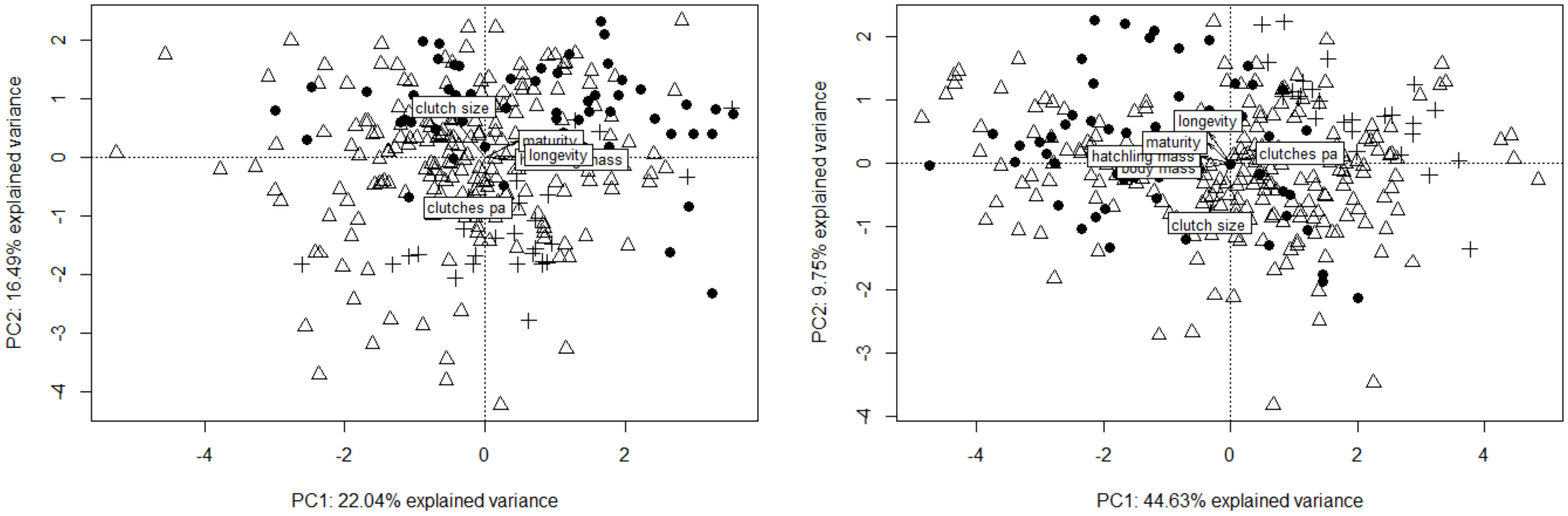

S2 Fig. Weight-adjusted principal component analysis (pPCA) with two global principal components (PC1/PC2) for life-history traits of 300 squamate species. Dataset is taken from Scharf et al. (2014). Instead of direct measurements of adult weight (g), Scharf et al. (2014) used length-weight allometries to calculate the body mass of squamate species from snout-vent-length (SVL, mm) or total length (TL, mm). Axes are based on life-history traits of species. Arrows indicate loadings, thus the contribution of life-history traits to PC1 and PC2. The phylogenetic weight matrix was taken from the phylogeny of Pyron et al. (2013). a) In this analysis life-history traits were analysed thereby correcting for body mass. Clutch size and clutches per year did mainly contribute to the PC2. This is due to the life history of the geckos, which lay 1 or 2 eggs but for multiple times per year (see textbooks on geckos: e.g. Rogner, 1992; Rösler, 1995; Bartlett & Bartlett, 2006). Loadings of PC1: body mass=0.43, hatchling mass=-0.24, clutches pa=-0.45, clutch size=-0.45, maturity=0.48, longevity=0.57; loadings of PC2: hatchling mass=-0.04, clutches pa=-0.68, clutch size=0.68, maturity=0.28, longevity=0.04. b) Body mass was added as further trait to life-history traits. Loadings of PC1: body mass=-0.47, hatchling mass=-0.48, clutches pa=0.39, clutch size=0.34, maturity=-0.38, longevity=-0.35; loadings of PC2: body mass=-0.07, hatchling mass=0.12, clutches pa=0.16, clutch size=-0.78, maturity=0.32, longevity=0.50. Clutches pa = number of clutches per annum (per year).
